# Electron Transport Properties in High Electron Mobility Transistor Structures Improved by V-Pit Formation on the AlGaN/GaN Interface

**DOI:** 10.1021/acsami.3c00799

**Published:** 2023-04-06

**Authors:** Alice Hospodková, František Hájek, Tomáš Hubáček, Zuzana Gedeonová, Pavel Hubík, Matěj Hývl, Jiří Pangrác, Filip Dominec, Tereza Košutová

**Affiliations:** † Institute of Physics CAS, v. v. i., Cukrovarnická 10, 162 00 Prague 6, Czech Republic; ‡ Faculty of Nuclear Sciences and Physical Engineering, Czech Technical University in Prague, Břehová 7, 11519 Prague 1, Czech Republic; § Faculty of Mathematics and Physics, Charles University, Ke Karlovu 5, 121 16 Prague, Czech Republic

**Keywords:** HEMT, GaN, AlGaN, metal−organic vapor phase epitaxy, dislocations, electron mobility

## Abstract

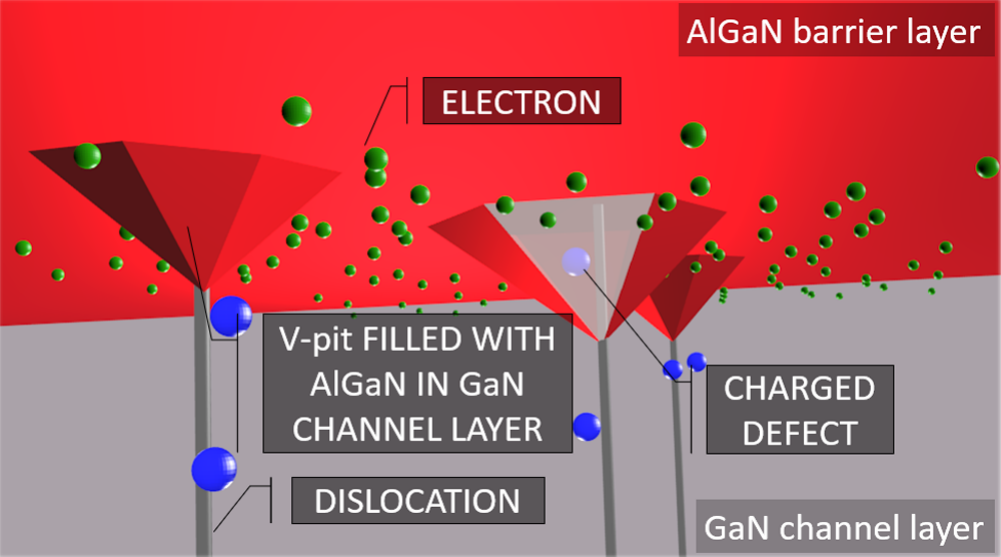

This work suggests new morphology for the AlGaN/GaN interface which enhances electron mobility in two-dimensional electron gas (2DEG) of high-electron mobility transistor (HEMT) structures. The widely used technology for the preparation of GaN channels in AlGaN/GaN HEMT transistors is growth at a high temperature of around 1000 °C in an H_2_ atmosphere. The main reason for these conditions is the aim to prepare an atomically flat epitaxial surface for the AlGaN/GaN interface and to achieve a layer with the lowest possible carbon concentration. In this work, we show that a smooth AlGaN/GaN interface is not necessary for high electron mobility in 2DEG. Surprisingly, when the high-temperature GaN channel layer is replaced by the layer grown at a temperature of 870 °C in an N_2_ atmosphere using TEGa as a precursor, the electron Hall mobility increases significantly. This unexpected behavior can be explained by a spatial separation of electrons by V-pits from the regions surrounding dislocation which contain increased concentration of point defects and impurities.

## 1 Introduction

In the last few years, a lot of attention of both research and industry has been devoted to wide-band gap materials such as GaN, SiC, or Ga_2_O_3_. The wide bandgap results in higher breakdown voltages and enables us to use the material for high-power applications with a simultaneous decrease of the device size and energy loss. SiC and Ga_2_O_3_ have significant advantages in native substrate availability, which is beneficial especially in vertical devices for high-power applications. SiC has additionally very high electron saturation velocity giving it some chance to compete with GaN. However, GaN is the best candidate in case that the combination of high frequency and high power is required.^1,2^ Besides outstanding material properties, such as high breakdown voltage, high electron velocity, and good thermal conductivity,^3,4^ GaN has one crucial advantage, polar wurtzite crystal structure with strong polarization electric field near interfaces.^5^ Thanks to this, the AlGaN/GaN heterointerface forms a quantum well (QW) with a 2D electron channel on the GaN side. In this channel, the sheet carrier density is typically in the order of 10^13^ cm^–2^ depending on the thickness and composition of the AlGaN barrier. This high carrier concentration in the channel helps to shield the electric field around ionized impurities and considerably decreases electron scattering on them.^6,7^ Thus, the achieved electron mobility in the 2D channel can be much higher than that in bulk GaN or in SiC, which is advantageous for high-frequency transistors. The combination of high carrier concentration and high electron mobility results in a low channel resistance and consequently in high cut off frequencies and lower energy loses. Thus, GaN-based high electron mobility transistors (HEMTs) are the most promising solution for the nowadays microwave communication industry, new-generation 5G and 6G cell phone networks, satellite communications amplifiers and TV broadcasting, or big data (cloud) storage centers.

However, there are still problems that must be solved. One of them is to increase the electron mobility in the 2D channel from usually obtained values, around 1500 cm^2^/(V s), closer to the values which are theoretically promised, above 2000 cm^2^/(V s). Another problem is to protect carriers from being captured by deep traps.^8^ Both problems deteriorate frequency properties of final HEMTs.

The widely used technology for the preparation of GaN channels in HEMT transistors is growth at a high temperature of around 1000 °C in an H_2_ atmosphere. The main reason for choosing this technology is to prepare a very smooth epitaxial surface for the AlGaN/GaN interface and to achieve the channel layer with carbon concentration as low as possible. In this work, we show that the smooth interface, surprisingly, may not be optimal for achieving the highest electron mobility in 2D electron gas (2DEG).

Some studies supposed that the scattering mechanism of carriers in 2D channels on dislocations is negligible and the dislocation density plays a minor role in transport property control.^9^ Another study^10^ shows that in the case of high dislocation density above 10^10^ cm^–2^ scattering on charged dislocations deteriorates transistor performance. In our previous work,^11^ we have shown that density of dislocations has significant influence on the electron mobility in 2DEG. Recently, it was demonstrated that improvement of crystal quality led to an increase in electron mobility in 2DEG, up to the theoretically predicted value.^12^ The mechanism of mobility deterioration by dislocations may include scattering on charged dislocations, rough interface, and point defects surrounding them. In this work, we show the possibility of suppressing the influence of dislocations on 2D electron gas mobility by a special design of the GaN channel using advantages offered by the formation of V-pits.

## 2 Experiments and Simulation

We prepared all studied structures by metal organic vapor phase epitaxy (MOVPE) on c-oriented sapphire substrates using an Aixtron 3 × 2” CCS MOVPE system, equipped with LayTec EpiCurveTT in situ monitoring. For the growth of GaN buffer layers and AlGaN barriers, trimethylgallium (TMGa), trimethylaluminum (TMAl), and ammonia (NH_3_) precursors were used, always in an H_2_ atmosphere. The GaN channel layer was grown either in an N_2_ atmosphere at 870 °C from triethylgallium (TEGa) or in a H_2_ atmosphere at temperature 1050 °C with TMGa used as the precursor, as will be explained later. Selected HEMT structures were prepared on two different types of templates, one with a lower dislocation density (LDD) of 9 × 10^8^ cm^–3^ and another with a high dislocation density of (HDD) 2.8 × 10^10^ cm^–3^, to compare the influence of dislocation density on transport properties of electrons in 2DEG. The difference in dislocation density was achieved by the design of the nucleation layer growth and subsequent coalescence of smaller (HDD) or bigger (LDD) GaN islands. More details about the LDD and HDD sample preparation can be found in ref (11).

Each examined HEMT structure was prepared on both types of templates in a single technological run to evaluate the influence of dislocation density on different structure parameters.

**Figure 1:**
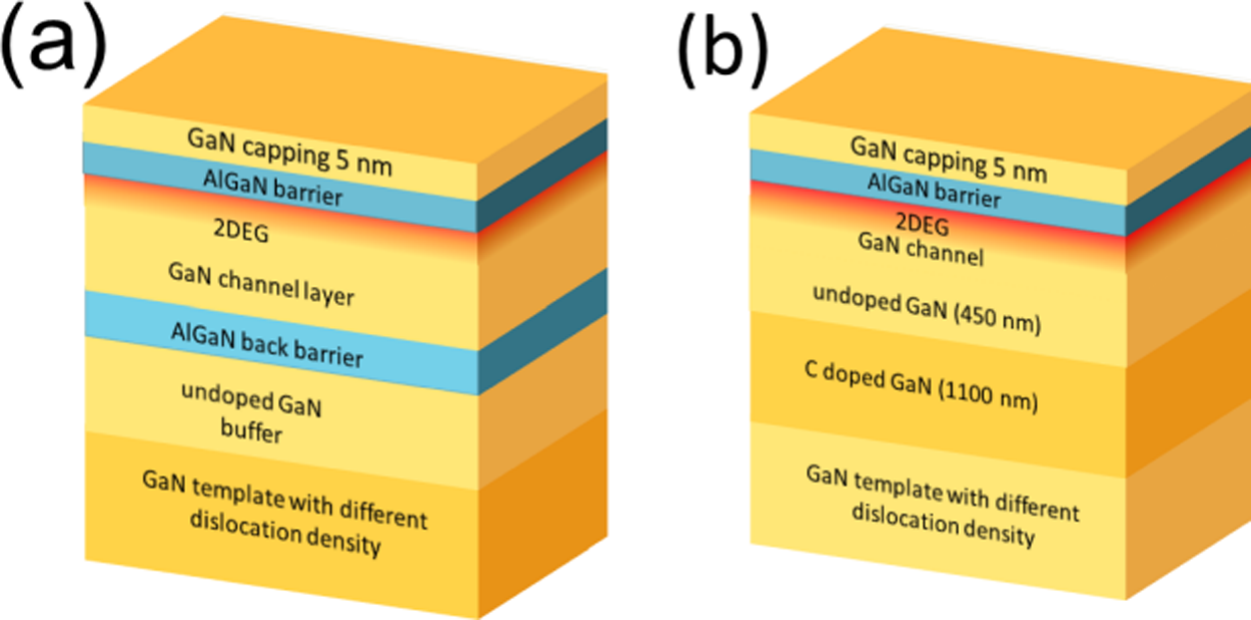
HEMT structures with (a) AlGaN back barrier and (b) C-doped GaN buffer.

Two types of HEMT heterostructures with different buffers preventing penetration of current to deeper layers^13^ have been prepared and are discussed in this work, one with an optimized AlGaN back barrier,^11^ see scheme in Figure 1a, and the other with highly carbon-doped GaN buffer, see Figure 1b.

All structures were characterized by resistance and Hall effect measurements, using the van der Pauw method on square (ca. 10 mm × 10 mm) samples with soldered In contacts in corners.

Secondary ion mass spectroscopy (SIMS) was provided by EAG laboratories for structures grown on LDD and HDD templates.

For all atomic force microscopy (AFM) scans, Bruker Dimension ICON AFM was operated in semicontact Peak Force QNM mode with Aspire conical force modulation (CFM) probes. The symmetrical shape and combination of small tip radius (guaranteed <10 nm) and sharp tip cone angle (30°) of these probes ensure true and symmetrical representation of all sample features. For processing the data, Gwyddion software^14^ was used.

The crystal structure was analyzed by X-ray diffraction measurements done by a Rigaku SmartLab diffractometer. Dislocation density was estimated from the (002) and (102) rocking curves, and the Al concentration was checked from the HRXRD θ/2θ scans of (002) diffraction.^15^ The layers were found to be pseudomorphically strained by mapping the surrounding of the (114) GaN diffraction peak in reciprocal space.

Simulation of the 3D HEMT structure was performed in nextnano^++^ software.^16^ The V-pit was modeled as an AlGaN inverted hexagonal pyramid covered by the AlGaN layer (10 nm) with the same Al content (24%). Sidewalls are formed by the plane {10–11} and their semipolar equivalents,^17^ and the AlGaN layers are assumed to be pseudomorphically strained to GaN layer. Surface potential is set by the Schottky barrier height (1.15 eV); no bulk doping was used. Results were obtained as a self-consistent solution of Schrödinger and Poisson equations. The number of grid points in *x* and *y* directions was set to 55 and in *z* direction [0001] 145.

## 3 Results and Discussion

### 3.1 Influence of Dislocation Density

In our previous work, we have proved that the dislocation density has significant influence on electron mobility in 2DEG.^11^ However, the mechanism of mobility deterioration by increased dislocation density was not answered yet. It could be caused by scattering carriers on charged dislocations, although scattering on dislocations was not previously considered as a significant mechanism in HEMT structures with 2DEG.^9,18^ The influence of interface morphology may also be responsible for lowering the electron mobility in structures with a high dislocation density.^18^ Another mechanism could be scattering on ionized impurities or carrier capture on traps surrounding dislocations.^19^

**Figure 2:**
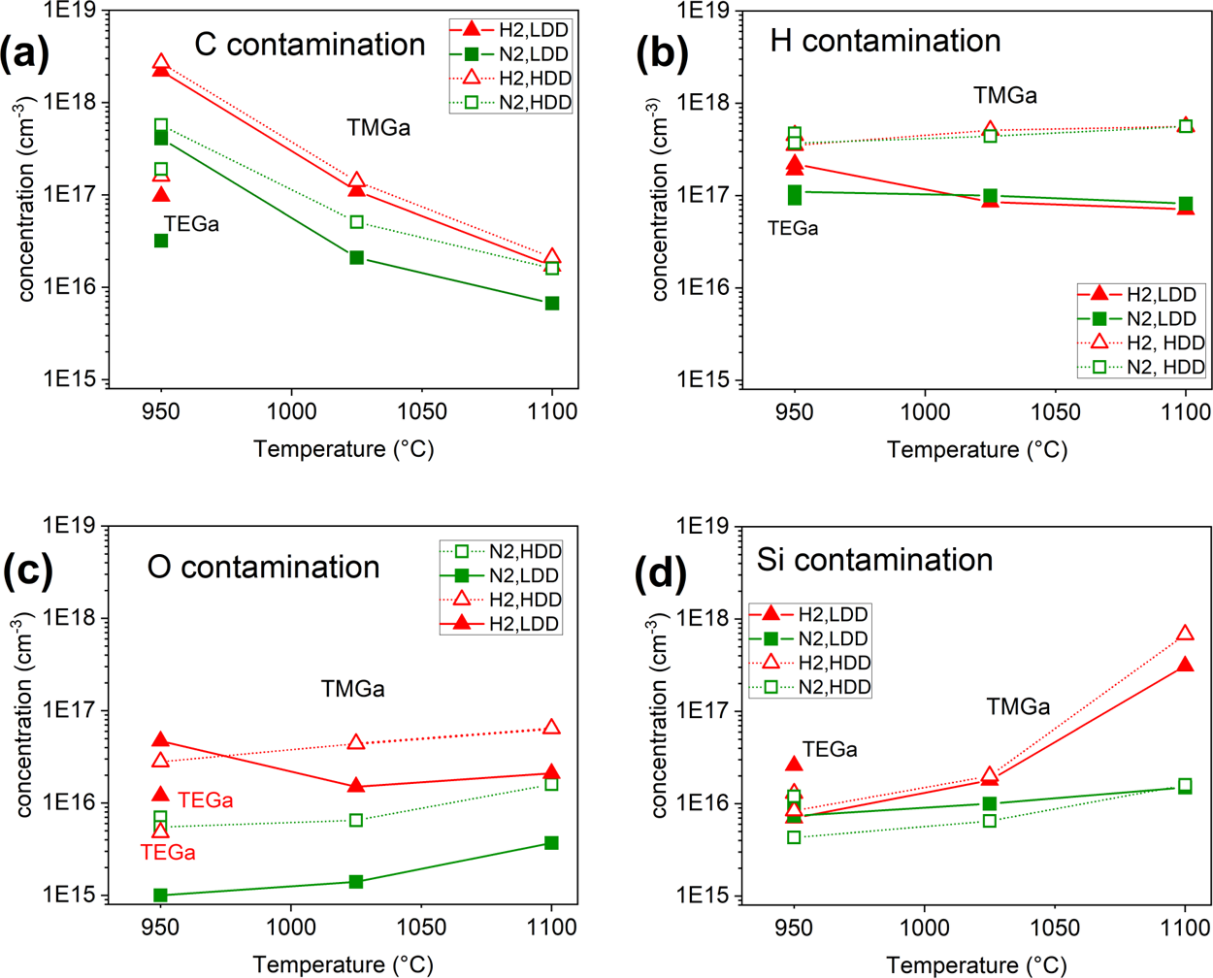
Concentration of impurities in GaN layers grown from TMGa at different temperatures and reactor atmospheres (N_2_, green symbols and lines and H_2_, red symbols and lines) in case of lower and higher dislocation density (LDD, solid symbols and HDD, open symbols): (a) carbon, (b) hydrogen, (c) oxygen, and (d) silicon contamination. For a temperature of 950 °C, contamination of GaN layers grown using TEGa is shown for comparison (points without line connection).

To prove the hypothesis that impurity incorporation is enhanced in the vicinity of dislocations, we have checked the concentration of four most common contaminants, C, H, O, and Si, in MOVPE prepared GaN layers by SIMS. The incorporation of impurities into MOVPE-grown layers is a very complex process which can be influenced by different growth parameters, such as growth temperature, type of carrier gas, or type of precursor. Carbon and hydrogen originate from precursors, while silicon and oxygen contamination is usually supposed to originate from silica liner etched by hydrogen and ammonia. Comparison of SIMS results obtained on GaN layers is shown in Figure 2a–d. Studied layers were grown at different temperatures, different carrier gasses (green symbols for N_2_, red symbols for H_2_), and on templates with different dislocation density (solid symbols for LDD and open symbols for HDD samples).

It can be noticed that incorporation of various contaminants depends on growth parameters and dislocation density in different ways. For instance, C contamination is decreased with temperature, while Si contamination is increased, especially in an H_2_ atmosphere. Two more general features can be observed. First, contamination is enhanced in a hydrogen atmosphere for all studied contaminants, except hydrogen itself. Increased contamination of GaN layers in the hydrogen atmosphere is in agreement with refs (20, 21). Second, concentration of all studied contaminants is higher in HDD samples with exception of silicon contamination, which seems to be independent of dislocation density. Increased carbon, oxygen, and hydrogen contamination in layers with higher dislocation density suggests that there is a region around each dislocation where incorporation of these impurities is enhanced. Since C and O incorporate on the nitrogen site in the GaN lattice, while Si on Ga sites, it could be a sign that around dislocation there is a higher probability of impurity incorporation to the nitrogen sublattice.

### 3.2 Protection of Carriers by V-Pits

**Figure 3:**
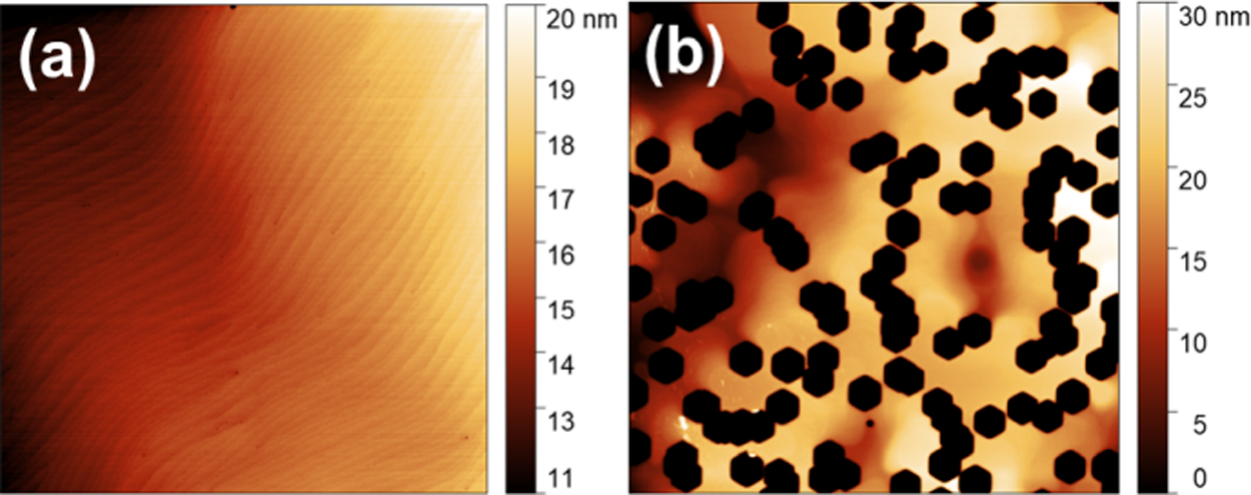
AFM surface images of sample (a) grown in a H_2_ reactor atmosphere at 1050 °C and (b) grown in an N_2_ reactor atmosphere at 870 °C.

V-pits are morphological defects formed around dislocations with screw components.^22^ They are formed at lower growth temperatures under an N_2_ reactor atmosphere (Figure 3b), while at higher growth temperatures and a hydrogen atmosphere, the surface is smooth, see Figure 3a. V-pits were recognized to be beneficial for enhancement of photoluminescence efficiency in heterostructures containing InGaN/GaN QWs.^17^ On their side walls, the InGaN QW is thinner, and so the V-pits serve as a barrier for electrons in InGaN QWs which separate them from the region around dislocation containing a higher concentration of point defects. The optimal size of V-pits was found to be 200 nm in diameter.^23^ However, this mechanism to suppress the influence of dislocation requires the presence of InGaN QWs.

Now, let us consider the AlGaN/GaN heterostructures used in HEMT structures. We have shown in Section 3.1. that contamination of H, O, and C is increasing with higher dislocation density. Thus, we can suppose that there is a region in the vicinity of dislocations with enhanced contamination by these atoms. A similar observation was also published in ref (24). Among these contaminants, carbon was reported as the most harmful defect with respect to HEMT frequency properties.^19,25^ Thus, it is important to protect electrons in 2DEG from being trapped by carbon defects around dislocations.

**Figure 4:**
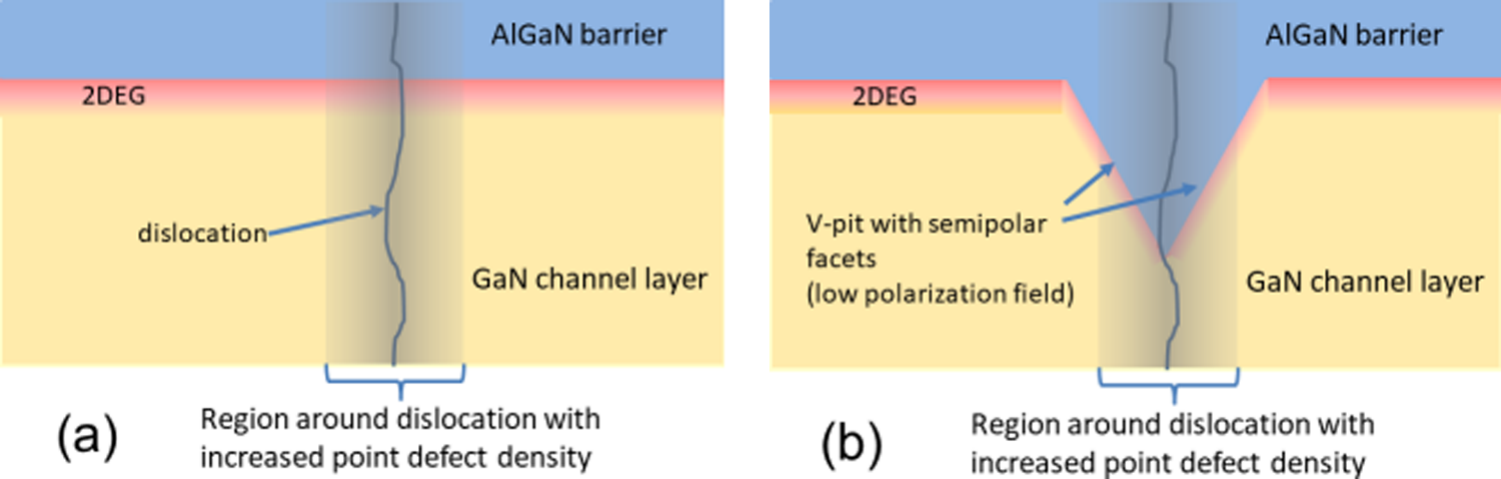
Schemes of the AlGaN/GaN interface cross section with dislocation. (a) Flat interface: region of increased point defect concentration penetrates to 2DEG. (b) Structure with V-pits: semipolar V-pit facets represent the barrier for electrons and prevent electrons to enter the region with increased defect density.

Surprisingly, in HEMT structures V-pits could also be used to suppress the influence of dislocations, although by different types of mechanisms. The mechanism of how the V-pit helps to separate electrons from the deteriorated region in dislocation vicinity is explained in Figure 4.

2DEG is formed near the AlGaN/GaN interface, where the lowest potential of conduction band forms deep triangular-like QW by band discontinuity and polarization field. The highest polarization field is on interfaces perpendicular to the [0001] direction. Without V-pits, 2DEG enters the regions with high point defect density around dislocations, see Figure 4a. However, if the high defect density region intersects a V-pit, the situation changes, see Figure 4b. V-pit facets have semipolar orientation; thus lower polarization field and much shallower triangular QW can be expected on the semipolar AlGaN/GaN interface. This is why, the shallow QW (having its potential higher than its surroundings) on V-pit facets could serve as a barrier for electrons in 2DEG near the flat AlGaN/GaN interface. In case the GaN channel layer would be prepared with V-pits on their surface and overgrown by the AlGaN barrier, electrons in 2DEG would be separated from the deteriorated region around dislocations similarly as in the case of InGaN QWs.

**Figure 5:**
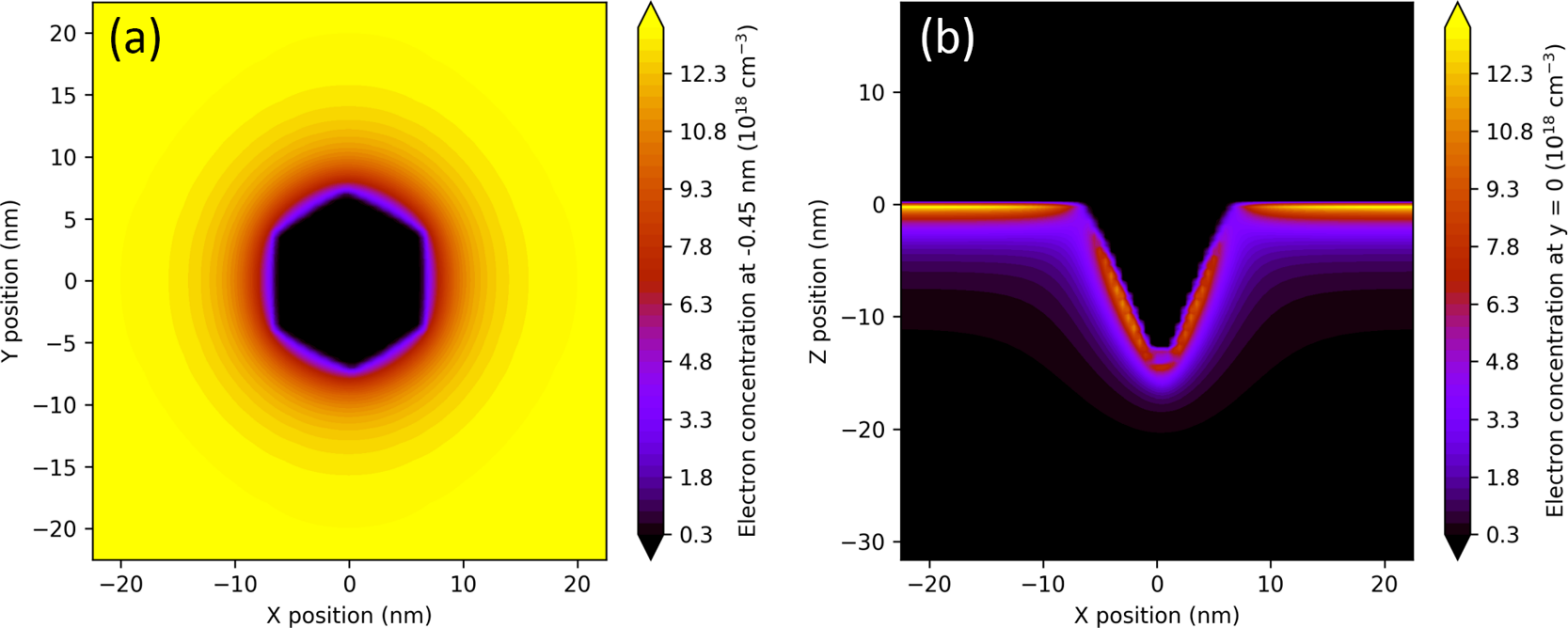
Electron density in the (0 00 1) plane in the 2DEG channel around the V-pit filled by AlGaN. Simulated by Nextnano (a) color plot of electron density in the plane of 2DEG 0.45 nm below the AlGaN/GaN interface, and (b) plot of electron density in the cross section through the V-pit.

Electron density in the (0001) plane in the 2DEG channel around the V-pit filled by AlGaN was simulated by Nextnano software, see Figure 5. It can be seen that the electron density near the V-pit is suppressed and that the 2DEG is avoiding the region near the V-pit.

To check this hypothesis experimentally, we have prepared HEMT structures with GaN channels with or without V-pits at the AlGaN/GaN interface. For structures without V-pits, classical technology was used; the GaN channel was grown from TMGa at 1050 °C under a hydrogen atmosphere with a smooth surface morphology. Under these conditions, a very smooth surface is obtained (see Figure 3a) with a relatively low carbon concentration in the channel layer, around 1 × 10^16^ cm^–3^ according to SIMS results. Different technology was used for the GaN channel with V-pits: GaN was grown from TEGa at 870 °C under an N_2_ atmosphere. HEMT structures with and without V-pits were prepared on both LDD and HDD templates. To obtain a complex picture of samples with V-pits, impurity incorporation and interface morphology, we have prepared special samples for SIMS and AFM measurement.

**Figure 6:**
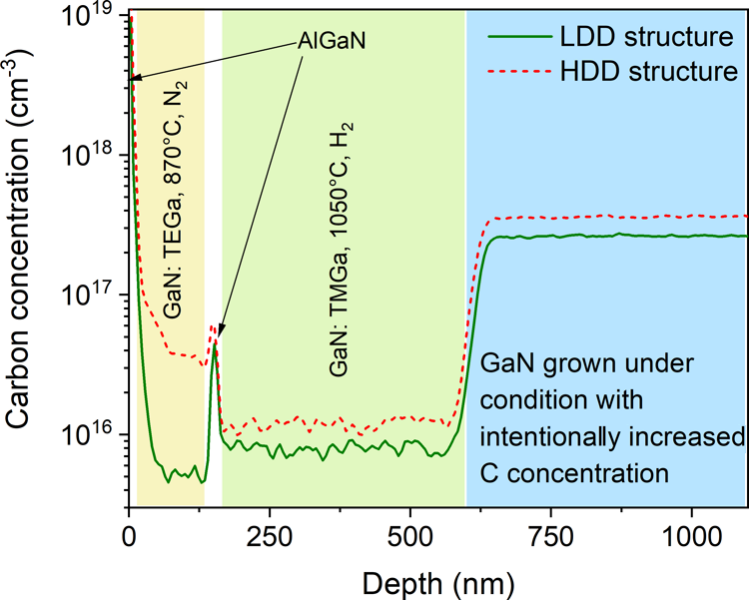
Comparison of carbon contamination in the HEMT structure prepared on high (HDD) and lower dislocation density (LDD) templates, measured by SIMS.

**Figure 7:**
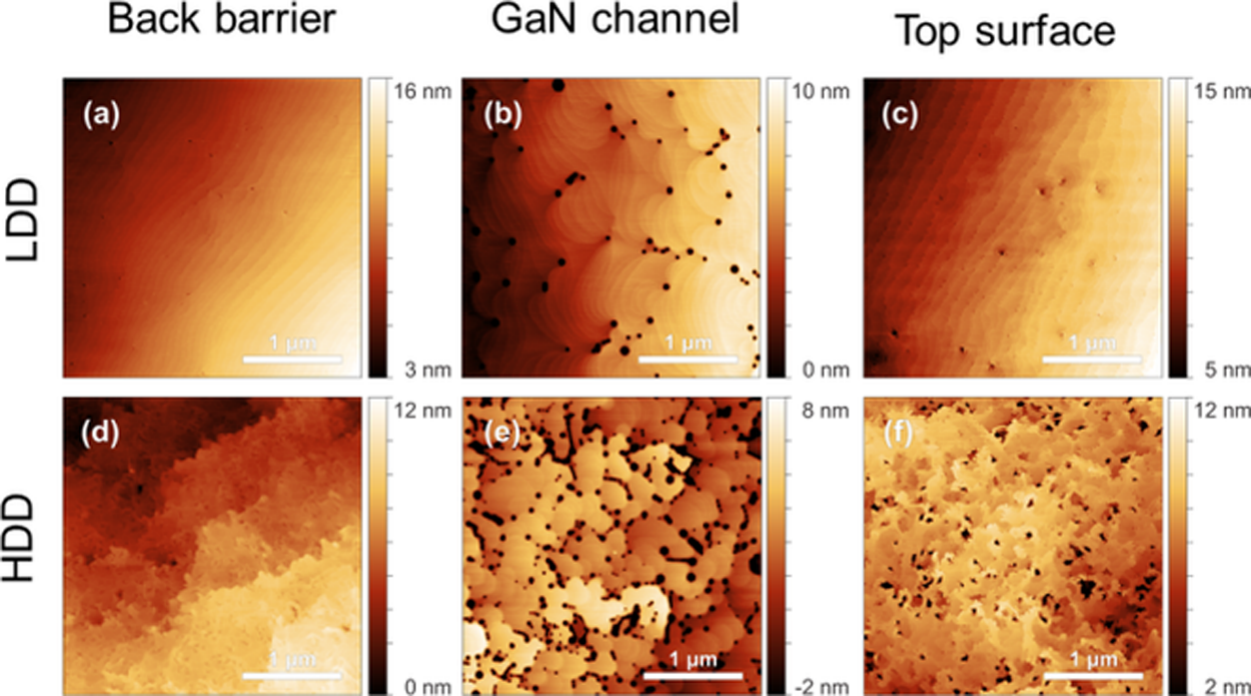
AFM surface images of samples stopped in different stages of growth process, prepared on LDD template (a–c) and on HDD templates (d–f). The growth was stopped after the AlGaN back barrier (a, d), GaN channel layer (b, e) and after the AlGaN main barrier layer (c, f).

First, we have checked carbon incorporation by SIMS in two HEMT structures prepared on templates with high and lower dislocation density, see Figure 6. These two samples contain resistive intentionally carbon doped buffer as well as an AlGaN back barrier; the GaN channel layer was prepared using TEGa as the precursor at lower temperatures (870 °C) and an N_2_ atmosphere to form V-pits. As expected, higher carbon concentration in all layers was measured for samples with higher dislocation density. Surprisingly, the highest relative difference in carbon concentration between HDD and LDD samples was found in the GaN channel. In addition, the channel layer of the HDD sample exhibits a significant increase of carbon concentration even with respect to the underlying layer. This could be caused by higher carbon incorporation on V-pit facets,^26^ which were formed during the growth of this layer and have a higher surface ratio in case of HDD samples. Higher measured carbon contamination by SIMS could also be an effect of rough surface of this sample with not completely overgrown V-pits shown in Figure 7f.

The morphology of different interfaces in HEMT structures was studied on stop-growth samples (one set prepared on HDD and the second on LDD templates) by atomic force microscopy (AFM). AFM images of surfaces of stop-growth samples in Figure 7a,d shows a surface morphology of the AlGaN back barrier, (b) and (e) the surface morphology after GaN channel growth, and (c) with (f) the morphology of surface after main AlGaN barrier growth. It is clearly visible that the dislocation density has strong influence on the surface morphology even in the case when no V-pits are formed, compare Figure 7a,d. AFM results shown in Figure 7b,e confirmed formation of V-pits, which decorated the threading dislocations with a screw component.^22^ From image (c), it can be seen that after 12 nm of AlGaN growth, V-pits are almost completely filled by AlGaN in case of the LDD sample. On the contrary, the surface of the AlGaN barrier on the HDD sample is very rough, Figure 7f.

For Hall measurements, three sample doublets were prepared to decide whether V-pits improve or deteriorate the transport properties of 2DEG. The structure parameters and technology for each doublet were the same; the only difference was in the technology of the GaN channel layer which was prepared with or without V-pits as described above. Structure parameters together with obtained Hall measurement results are summarized in Table 1.

**Table 1:** Sample Parameters of Sample Doublets Prepared with and without V-Pits on the AlGaN/GaN Interface

sample	GaN channel morphology	buffer technology	AlGaN thickness (nm)	Al_*x*_Ga_1–*x*_N composition (*x*)	dislocation density (cm^–2^)	2D hall concentration (cm^–2^)	hall mobility (cm^2^/V s)
347H	flat interface	GaN:C	15	0.27	3 × 10^10^	1.25 × 10^13^	876
349H	V-pits	GaN:C	15	0.27	3 × 10^10^	1.31 × 10^13^	1034
551L	flat interface	AlGaN BB	11	0.24	9 × 10^8^	1.64 × 10^13^	1490
544L	V-pits	AlGaN BB	11	0.24	9 × 10^8^	1.35 × 10^13^	1754
551H	flat interface	AlGaN BB	11	0.24	3 × 10^10^	9.50 × 10^12^	765
544H	V-pits	AlGaN BB	11	0.24	3 × 10^10^	8.18 × 10^12^	1123

The first doublet (samples 347H and 349H) was prepared on the HDD template with carbon-doped GaN buffer according to the scheme in Figure 1b. The second (samples 551La and 544La) and third (samples 551H and 544H) doublets were prepared with the AlGaN back barrier (Figure 1a) on LDD and HDD templates, respectively. It can be seen that in all cases, using the technology with V-pit formation resulted in higher Hall electron mobility of 2DEG. Mobility was improved despite the fact that such an interface is much rougher in comparison to the technology when the H_2_ atmosphere was used. Surprisingly, in the case of both HEMTs grown on HDD templates, higher mobility was obtained on samples with the V-pit channel than with the flat channel, although these samples have higher carbon contamination of the channel layer according to SIMS results, see Figure 6 (compare carbon contamination in GaN: TEGa, 870 °C, N_2_ layer with GaN: TMGa, 1050 °C, H_2_ of the HDD sample). These results all together confirm that the V-pits spatially separate electrons from dislocation vicinity with increased concentration of point defects and impurities.

**Figure 8:**
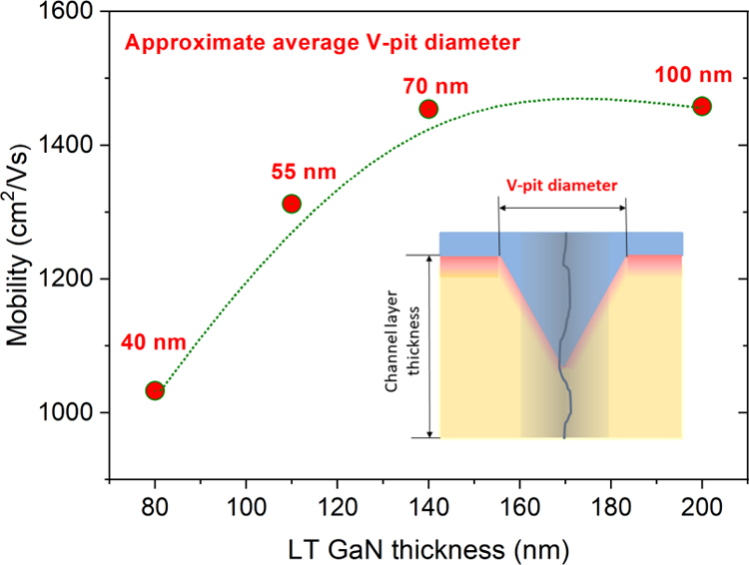
Dependence of Hall electron mobility on the thickness of GaN channel, which influences the V-pit size.

For optimal separation of electrons from the deteriorated region around a dislocation, the V-pit size can play an important role. The size of V-pits depends on the thickness of the GaN layer prepared in an N_2_ atmosphere. To find an optimal V-pit size, the set of samples with different GaN channel thicknesses was prepared on the LDD template. The dependence of Hall mobility on the GaN layer thickness is shown in Figure 8. It indicates that the sufficient thickness of the GaN channel layer is above 150 nm. At this thickness, the V-pit diameter is around 80 nm. It is much less than the optimal V-pit size for luminescence applications, which was found to be around 200 nm.^23^ The reason could be that luminescence is much more sensitive to defect concentration than electron mobility in 2DEG. There is also a tradeoff between the sufficient V-pit size and area of the flat interface with 2DEG.

## 4 Conclusions

Enhanced concentration of point defects, namely, carbon, oxygen, and hydrogen in GaN layers was proved by SIMS measurements for samples with increased dislocation density. Considering this fact and our observation that HEMT mobility is lower in the structures with high dislocation density, we have deduced that the scattering on point defects or capture of carriers in traps decorating dislocations is likely the main factor controlling the mobility in the channel layer.

We have shown that the atomically flat AlGaN/GaN interface is not required for obtaining high electron mobility of 2DEG in the HEMT structure. On the contrary, the formation of V-pits at the AlGaN/GaN interface might be beneficial for HEMT structures containing threading dislocations and leads to increased electron mobility, as measured by the van der Pauw method. V-pit morphology was obtained by changing GaN channel layer growth conditions. The high temperature growth in the H_2_ atmosphere was replaced by the growth at a temperature of 870 °C in an N_2_ atmosphere using TEGa as the precursor. The V-pits on the AlGaN/GaN interface spatially separate electrons from the regions surrounding dislocations, preventing the electron from being captured and/or scattered by impurities in dislocation vicinity. The mechanism of this improvement was confirmed both by simulation and experimentally. The optimal V-pit diameter was found to be around 70 nm. The electron mobility of 1754 cm^2^/V s with the carrier concentration in 2DEG 1.35 × 10^13^ cm^–2^ was obtained on samples with V-pit morphology of the AlGaN/GaN heterointerface prepared on the template with a dislocation density of 9 × 10^8^ cm^–2^.

## Abbreviations

HEMT: high electron mobility transistor
MOVPE: metal organic vapor phase epitaxy
2DEG: two-dimensional electron gas
TMGa: trimethylgallium
TMAl: trimethylaluminum
TEGa: triethylgallium
LDD: lower dislocation density
HDD: higher dislocation density
XRD: X-ray diffraction
HRXRD: high-resolution x-ray diffraction
AFM: atomic force microscopy
SIMS: secondary ion mass spectroscopy
